# Building a planter system using waste materials using value engineering environmental assessment

**DOI:** 10.1038/s41598-022-05300-0

**Published:** 2022-02-11

**Authors:** Fawaz S. Al-Anzi

**Affiliations:** College of Engineering & Petroleum, Kuwait University, PO Box 5969, 13060 Safat, Kuwait

**Keywords:** Environmental sciences, Hydrology, Engineering

## Abstract

Environmental challenges are significant threats to the planet; most of them are human-made hazards. Researchers are studying various environmental threats and trying to flourish sustainable policy for protecting the environment from various challenges worldwide. In Kuwait, researchers are paying attention to these various challenges and trying to reduce these issues in the most effective, economically innovative, and localized ways. Desertification and lack of water are the major significant examples of natural challenges faced by the environment. The fundamental goal of this study was to propose and implement a more cost-effective and economical alternative to the commercial Waterboxx kits technology. In this proposed work, the research team rebuilt a new prototype based on Value Engineering, whose functionalities are homogeneous to the most popular Waterboxx kits technology. Unlike the Waterboxx kits method, the new proposed framework decreased operational and capital expenditures and reduced the complexity of development and implementation by regular farmers. Since recycled plastic sheets and used tires are employed in the new method as grist, this method helps us fight against desertification and provide a better way to handle the ever-growing massive dumpsters of tires in the region of Kuwait; hence it helps us in getting rid of hazards due to the tire fires and bring in a more safe and friendly environment. A better substitute has been identified concerning the value from various substitutes considered for developing the prototype using a thorough examination with the help of the Function Analysis System Technique (FAST). A prototype of the proposed method was constructed and tested in a controlled lab atmosphere followed by an actual environment. Analysis of both soil and water on the experiment site was performed before and after the proposed prototype testing for conducting a cross-comparison of soil. This evaluation was performed to ensure the method we fabricated and tested is an effective environment-safe model. The simulation and analysis of the proposed method are very effective as the already existing model reduced and saved the cost of implementation. The cost reduced by the new proposed VE method than the already available model was 43.84% without paying attention to the intangible costs related to another environmental challenge, recycling waste materials that may also build up the cost-saving. This study illustrates how the proposed Value Engineering-based model became the foremost baseline method for developing a new innovative model to reduce cost and patentable design.

## Introduction

**Figure 1:**
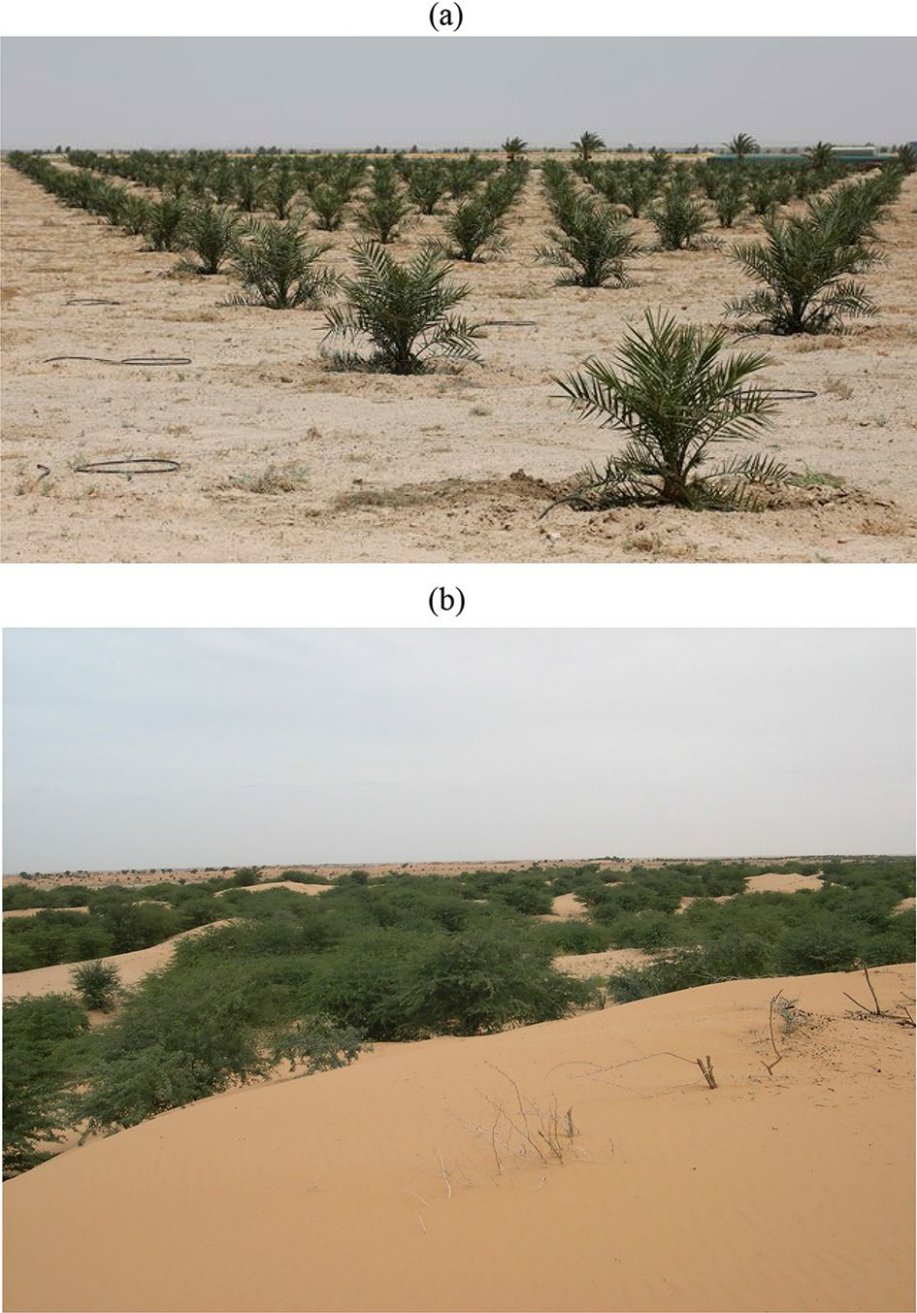
Fighting desertification: (**a**) Anti-sand shields in Iraq, south of Baghdad, (**b**) “Greenbelt” of Nouakchott, Mauritania.

Nowadays, the world is severely affected by severe environmental hazards. Similar to all other countries, Kuwait is also suffered from environmental challenges. One such environmental hazard faced by Kuwait is desertification, a category of land degradation. Desertification mainly takes place in dryland regions where it gradually becomes infertile, naturally dropping its water form, wildlife, and vegetation^1–3^. Reckless human activities on change in climate are the few main factors that intensify desertification. It became one of the most significant environmental and ecological challenges worldwide^1^. In order to fight desertification^2^, effective administration and maintenance are needed among tremendous investments and scientific methods for a more considerable period of time, as shown in Fig. 1.

One of the most acute challenges faced in Kuwait is water scarcity. Almost 95% of the pure water needed for Kuwait is gleaned through a highly expensive desalination method to the seawater^4,5^. Consumption of clean water is estimated to be considerably large due to the rapid population growth, which forced us to make available a massive amount of clean water with the given plant power. Kharraz et al.^6^ studied the desalination position and shortage of water very recently. Their study formed a list consisting of significant environmental factors that have an impact on desalination. They also considered the jeopardies related to the CO2 release, scorching saline water banishing (expulsion), and post the intakes of plants to creatures in the sea. As a result, it may be very controversial that the sustainability of desalination from seawater. Modern literature has various thoughts related to the issues in environmental, agricultural, and hydrological areas. You can find the detailed description from^7–20^.

Value engineering analyzes functions and systems in a disciplined and cooperative manner to attain intended outcomes at the lowest possible cost. Rad and Yamini^21^ conducted a study with a goal to present the principles and operational procedure of value engineering in building projects in a concise manner. According to the findings of this study, value engineering may be utilized to face adversity and complications of civil designs from the commencement of surveys to the completion of planning, building, profiting, and sustaining processes. In addition, their study looked into traditional techniques of measuring and evaluating functions of the project and compared those to value engineering as a means of improvement projects. As per their research findings, it is expected to achieve the objectives of the project while spending the lowest costs and guarantee the effectiveness of investment in the building projects management industry, which is a significant challenge of development plans in third-world countries, by using engineering in acceptable time periods and phases.

The construction industry is focused on meeting the demands of its clients by delivering projects that fulfill their goals and expectations on time, on budget, and as specified. Despite the government’s numerous housing programs, the poor continue to face a severe housing shortage. This goal will be met by combining the ideas of Value Management (VM) and Risk Management (RM) into the development of low-cost housing projects. Muhammad^22^ analyzed the effects of value engineering in the construction industry of Egypt. He also investigated the low-income housing projects of the government of the United Arab Emirates (UAElow-income)’s housing developments in Musaffah’s commercial district. Finally, this research recommended these low-income housing projects for the poor to different vendors such as the government and other professions or firms in this industry.

Kosala and Karunasena^23^ conducted a study on the present situation and actual use of the VE technique in the Sri Lankan construction industry and to make suggestions to construction firms and national construction regulatory authorities on how to standardize VE practices in order to achieve value for money for all stakeholders. An extensive literature study was conducted, and seven case studies, thirty-nine interviews, and six expert interviews across construction experts with deep knowledge of VE technique in the Construction Sector for gathering information. The study’s findings indicated insufficient building practitioners’ application, expertise, and experience with the VE approach. Reducing the contractor’s design responsibilities, providing a proper VE guideline, and restricting the VE process by law are only a few suggestions.

Various researchers conducted numerous works on different places, such as Rachwan et al.^24^, Ilayaraja and Eqyaabal^25^, Yan^26^, and so on for investigating and evaluating the effectiveness and importance of Value Engineering methodology for various disciplines. The benefit of VE was observed to be significantly greater when interdisciplinary engineering teams were included, which would also impact the design team, as is typical in the building.

In this research, we develop an innovative plan that is an alternative irrigation plan for recycling common waste add resisting desertification. Our alternative design is more convenient as well as cost-effective for employing in the environment of Kuwait. The parallel goals which were tried to achieve success in this study are listed below:

- Prepare an excellent design to utilize tire dumpsters mounted in Kuwait in an environmentally favorable manner.
- Identifying an economical substitute model for the commercially available Waterboxx model for cost reduction.
- Water resources optimization and collecting mist, rain, and haze water.
- Familiarizing and practicing farmers to use cutting-edge technologies to plant the trees and shrubs in the country’s farms.

## The main concept

The proposed model concentrates on becoming a better alternate for one of the most popular Groasis Waterboxx kits^8^. For erecting the proposed model, critical raw materials employed are recycled plastic sheets and used tires. Therefore, this new model opens an effective way to handle continuously increasing tire dumpsters in Kuwait and avoid the perils such as tire fires in a more secure, nature-friendly manner and the rationing of water for irrigation.

A dependent and organized model such as Value Methodology (VM)^5,6,27^ boosts procedures, products, and services. VM admits obtaining balance among needed characteristics, scope, safety, and performance, having value and additional sources required to achieve the clients’ requirements by the cost of balancing for accomplishing the tasks needed. VM concentrates on optimizing the objective function:

$${\text{Value}} = {\text{Function}}/{\text{Cost}}$$

- Value is known as the trustworthy accomplishment of the ability to meet client needs with a much lower standard rate.
- Function is known as the common trait of services or products.
- Cost is referred to as the total disbursement needed to furnish structure, service, system, or product.

**Figure 2:**
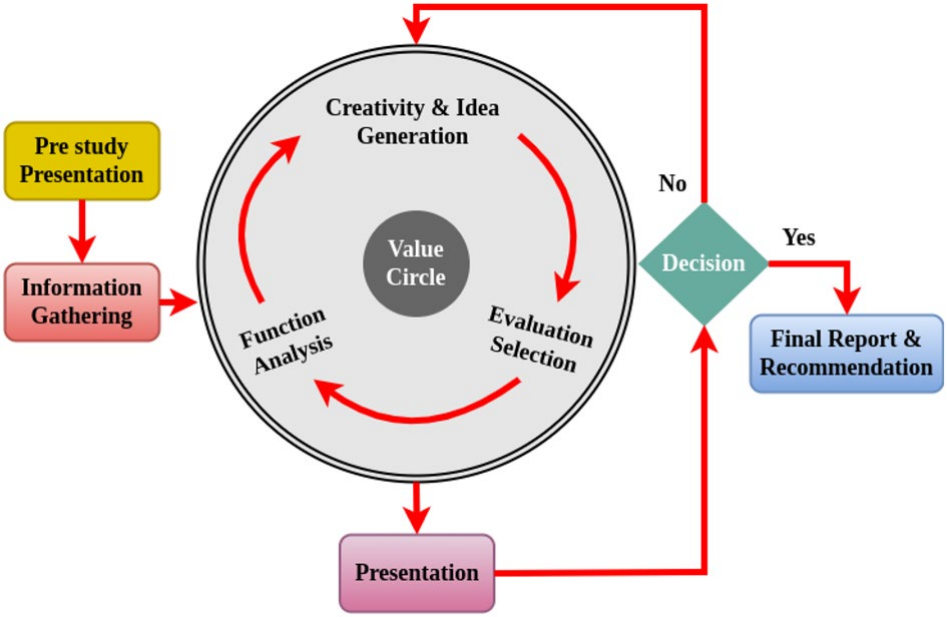
The seven phases in value engineering methodology.

The general activity plan of VM provides a structural and systematic way, and it has seven phases, as shown in Fig. 2. They are the following:

1. Information Gathering Phase: Collect all the required information to figure out the task.
2. Function Analysis Phase: This phase is performed to analyze the project for clarifying and capturing all the particularized features and functions.
3. Creativity and Idea Formation Phase: This phase focuses on formulating all the expedient and practicable ideas or concepts and their substitutes needed to carry out the required facilities.
4. Evaluation and Selection Phase: Evaluate various ideas or thoughts and elect the most viable one.
5. Development Phase: Pay attention to enhancing cost and design by selecting and combining exceptional opportunities.
6. Presentation Phase: Advice the customers and stakeholders regarding cost.
7. Implementation Phase: Implementing and following up the value team’s recommendations.

Implementing VM procedure with the help of a multi-disciplinary team contributes more effective results. A Certified Value Specialist should lead the VM team in helping to make accurate interventions with the value management and practices. The specialist must be licensed from SAVE International^®^^28^ to guide such a team. Through the global spectrum of industries and agencies, VM is embraced for building contractors and designers, pharmaceutical corporations, chemical processors, automobile producers, etc. The significant benefits of the employment of the VM model in the organization are it acts as both a well-established cost reduction and value improvement medium. The VM helps the organizations accomplish savings in cost, time, and better contribution development to boost competitive functions.

The primary goal of this research is to provide a design substitute for a stand-alone plants’ irrigation model for fighting desertification which is both cost-reducing and economical. VE can be referred to in more detail from^28–30^ if any reader needs it. The formal definition of Value Engineering can be summarized as “an organized team effort aimed at analyzing Functions and Quality of projects (goods, services, and processes) in order to generate practical, cost-effective alternatives that meet customer requirements.” Since the quality can be improved with this model, it cannot be considered simply a cost reduction tool. Another declaration about Value Engineering by John D. Sankey and Merle L. Braden Office of Value Engineering, U.S. Army Corps of Engineers, is that “Value Engineering has great potential in hazardous, toxic, and radiological waste remediation. Environmental work is usually of high cost. It offers greater opportunities for VE savings.” Environmental service providers must maintain the quality to cling to the compliance certification, including costs to guarantee viability^31,32^.

The key focus of the VE procedure while inflating the environmental solutions’ performance to satisfy the public needs is enhancing the staff creativity, effectiveness and disposing of waste, and reducing the costs of analysis, investigation, constructions, operations, and future maintenance. Organizations, both governmental and industrial, must be faced with increasing pressures due to the strict environmental rules issued by various countries. Cost-effective solutions that are both effective and safe must be delivered by them.

- Quick, creative, effective solutions
- Optimized environmental impact
- Maximized resources
- Optimized construction expenditures
- Reduce life-cycle costs
- Identifying Alternative technology

Alternatives for environmental solutions are analyzed for judging value indices that embody cost and quality-related issues in the solution using these seven phases of Value Engineering.

## Functional analysis

Functional Analysis System Technique (FAST): The VE team identified the fundamental characteristics of design and its constituents during this phase. Every constituent or component is analyzed to recognize its objective and foremost reason and is listed. The vital role of this phase is to analyze the quality of the actual design, identify its mapping deficiencies, and generate a team to develop proper ideas for mitigating these issues, embellishing the appearance of the model, and enhancing the quality. A FAST diagram is composed to illustrate the functions and features (both primary and secondary) of the actual design components. VE engineering methodology is distinguished from all comparable procedures in this phase.

FAST can be employed to define, analyze, and utilize the know-how logic of design functions, how the correlation of these functions, and which of them needs curiosity if the project cost is to be enhanced. The functions of the Value Management system are identified by raising the question, “What does it do?” and many functions are contained in all strategies, processes, and designs. From which, design functions are figured out primarily. It will be evident that these design functions have one significant basic function during this model. Subsequent evaluations and classifications are performed due to this reason. The methods used to define and classify the properties are explained below:

Essential functions are the primary characteristic of service or products, and in rare cases, they contain more than one primary characteristic. However, it should be ensured that no two of these essential functions are mapped to different components. One most straightforward fundamental feature has been for most designs as per the rule. The underneath steps must be followed to complete the evaluation of Function:

1. Identifying and listing functions are performed by raising the “What does it do?” question. Each feature is represented as a “measurable noun” and an “active verb.”
2. Recognize the Basic Function(s) after identifying the functions which are all part of the component, and all other functions are viewed as Secondary Functions. Unwanted or aesthetic functions are identified from secondary functions.
3. The fundamental feature for the primary function is attained by raising “Why?” A higher-order function may be the solution. Even if the higher-order function is not in the scope of this research, it must be crucial to understand the research domain for the research team.
4. Specification, which provides meanings, functions, and identification, can be Specific and General. Specific represents for requirements and constraints of the design, and In General, fundamental limits yield from the design concepts. Exact quantities are encouraged to be present if it is possible.

**Figure 3:**
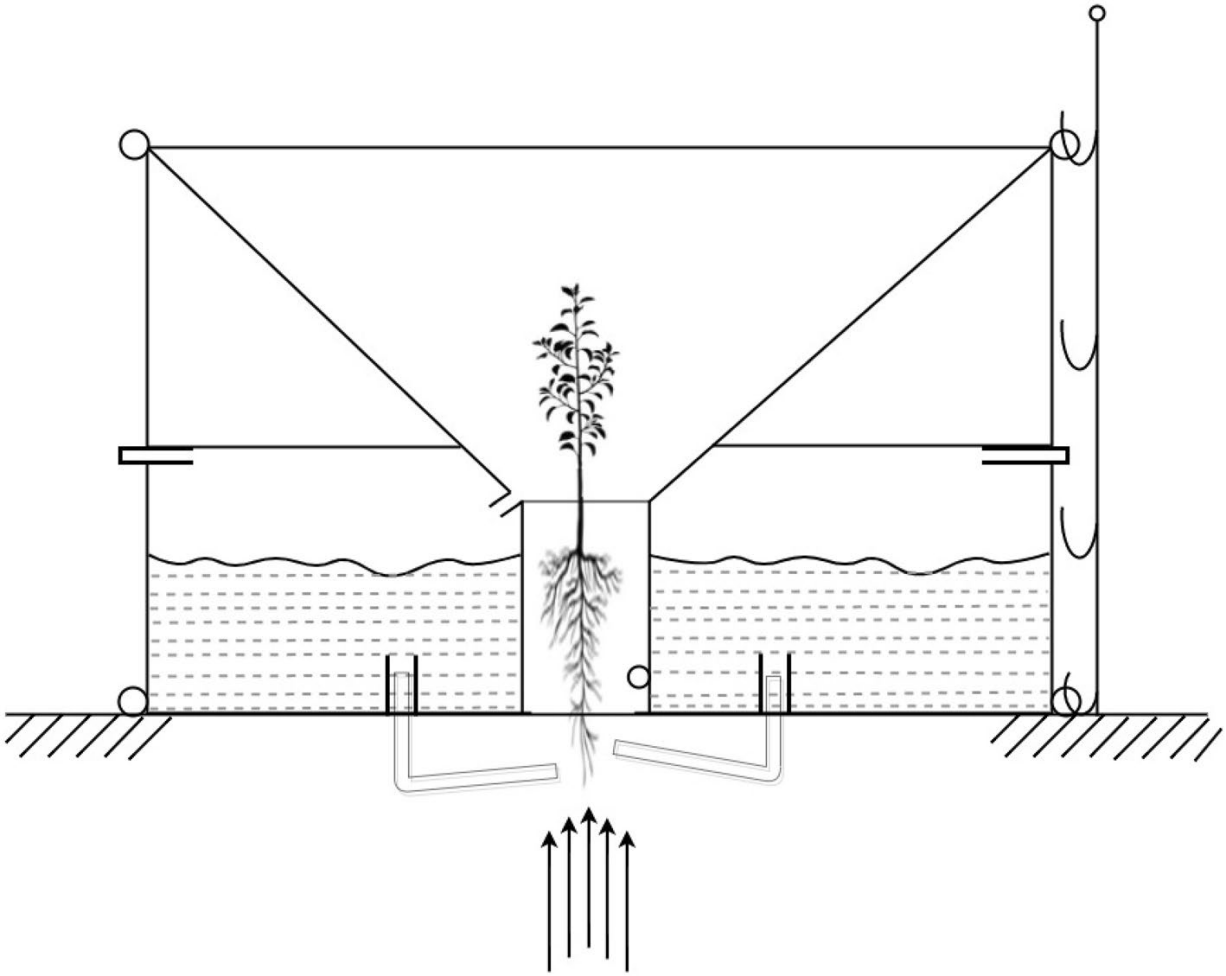
The original design of the WaterBoxx that the function analysis started with.

**Figure 4:**
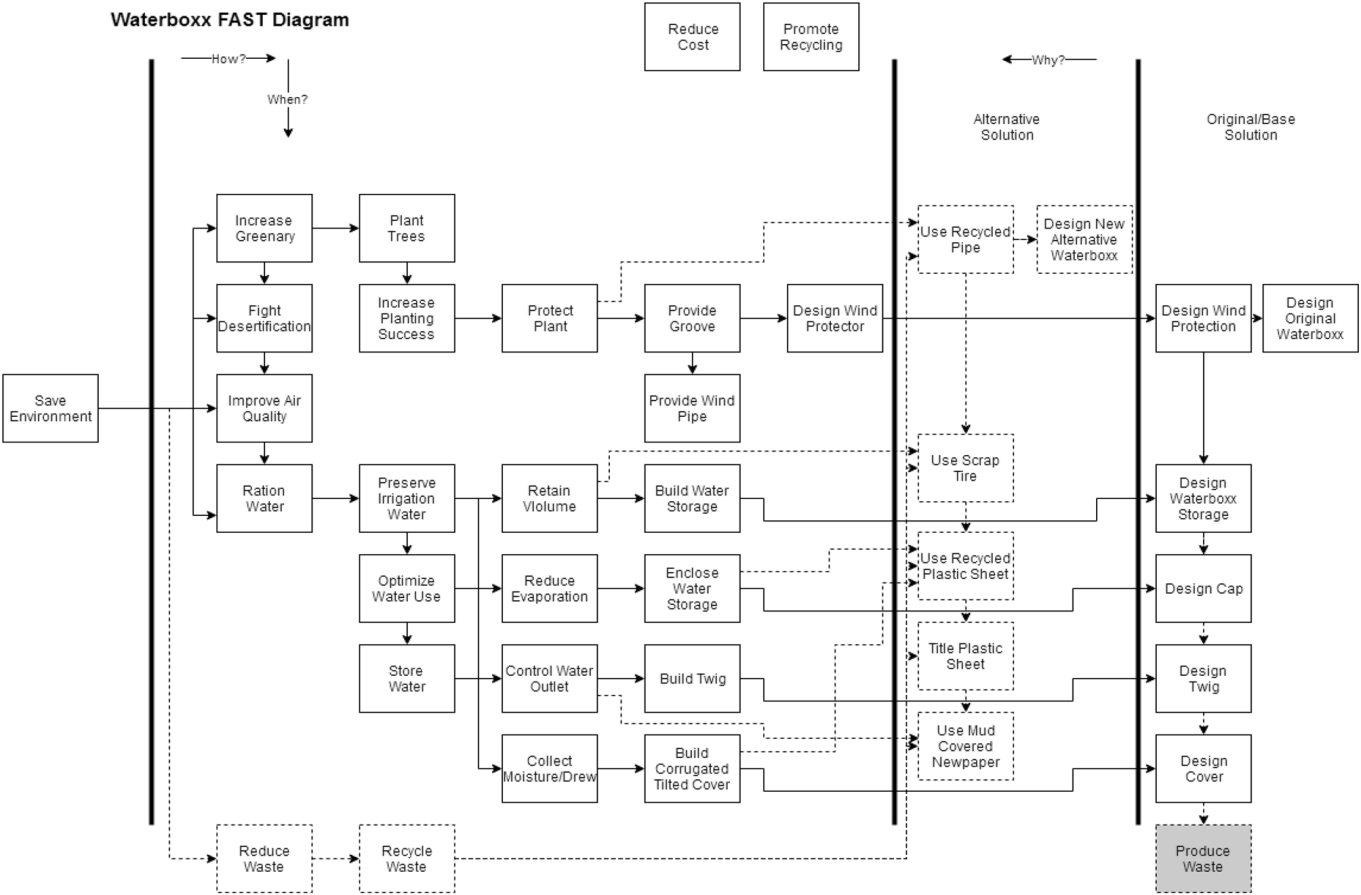
The constructed FAST Diagram of the WaterBoxx Designs.

The actual design of the Waterboxx model, as illustrated in Fig. 3, has been transformed to FAST as followed after the seven phases of the Value Engineering procedure as depicted in Fig. 4. This was accomplished by asking the How-WHY? Logic” and also finished the identification of primary feature. The abstraction stage of Functions to Lower Order from Higher Order is constructed to solve these questions.

The dashed box of the FAST diagram, as illustrated in Fig. 4, assisted the VE team to flourish the new ideas for enhancing the model design employing the components that extra environmental friendly and know-how WaterBoxx’s functional design.

## The alternative design

The team members of value engineering mostly interrogate two things when going through the production phase of the proposed alternate model: What are the available substitute ways to meet the specification? What else will carry out coveted features and functions?

Evaluation of various design alternatives will be performed based on its ability to meet crucial factors and conserve the overall cost as far as possible. The critical evaluation metrics used here to evaluate the models are the following.

**Figure 5:**
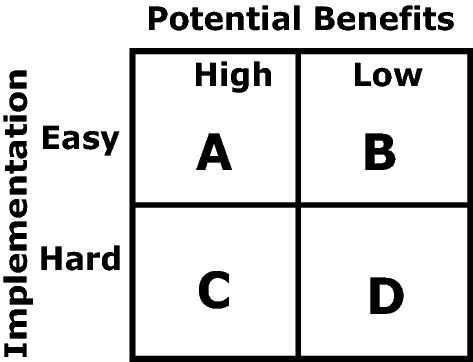
ABCD ranking method.

- Pre-evaluation: Pre-evaluation is performed to discard ideas that are impractical and impossible to implement.
- ABCD Ranking: The ideas that are possible to implement and practical to use are classified into various classes, as illustrated in Fig. 5. Ideas from squares A, B, and C will be scrutinized from these four classes. The ideas from A and B are fully considered, whereas only a few thoughts from C are taken to account.
- Weighted Evaluation Matrix: Based on diverse evaluation criteria, each others’ thoughts or a set of thoughts are evaluated using this technique. It needs to be enhanced due to the multi-objective behavior of the product or service’s quality axioms. We can identify two or more conflicting performance criteria with each other from those axioms. Hence, it is crucial to estimate the weights of each criterion. An easy way to determine the weights is to employ a weighted evaluation matrix for comparing the weights with each other.

### Focus points

The question “What does it do?” is raised for identifying the functions in Value Management. All strategies, procedures, and designs are associated with many functions. The foremost task of the team is to identify the functions and the capabilities which optimize the project cost. These functions and capabilities in the design hold various importance and ranks during this process. Considering this situation, these functions and capabilities must be reviewed and classified, significantly impacting the design^29^. VE Process mainly used two techniques; the Pareto law and Deficiency in Quality Profile.

### The Pareto law

**Figure 6:**
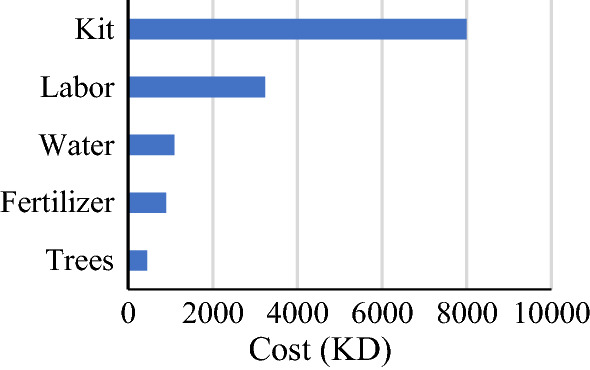
*Pareto’s Law* of the Cost Waterboxx.

*The law declares that the 80/20 rule* = 80% of the entire cost comprises 20% of the total items. The product or service components significantly impact the project cost and are recognized by defining and categorizing the functions using this Pareto law. The estimated Cost in Kuwaiti Dinars using normal Waterboxx for planting 1000 trees within three years is listed in Table 1, and Fig. 6 graphically illustrates the same.

**Table 1:** Cost of items in Waterboxx for three years life span.

Item	Cost (KD)
Trees	450
Fertilizer	900
Water	1095
Labor	3240
Kit	8000

### Second technique: the deficiency in quality profile

**Figure 7:**
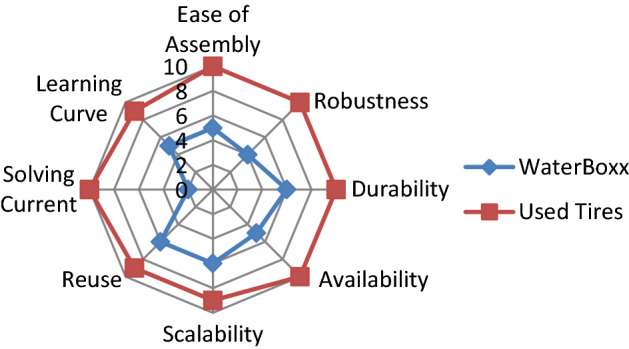
Quality profiles of the original design (in red) and proposed alternative design (in blue).

This model examines and determines the constraints for the actual design’s quality and proposes a substitute design to define and categorize the functions. Both designs’ quality profile is illustrated neatly in Fig. 7 and Table 2. In the alternate design, attention needed potential design flows are identified by the Profile’s quality deficiencies.

**Table 2:** Quality profile of original and proposed designs.

No	Criteria	Waterboxx	Used tires
1	Ease of Assembly	5	10
2	Robustness	4	10
3	Durability	6	10
4	Availability	5	10
5	Scalability	6	9
6	Reuse	6	9
7	Solving Current	2	10
8	Learning Curve	5	9

### Creative idea list

A few innovative thoughts developed all over the creativity/improvement phase of the VE method are presented in Table 3, which all are ranked using the ABCD ranking method.

**Table 3:** Creative ideas were generated for the proposed designs, impact, and ABCD categorization.

#	Idea description	Implementation easy/hard	Impact high/low	Category
1	Use recycled pipes to protect the plant	Easy	High	A
2	Use scrap tire to retain volume for water storage	Easy	High	A
3	Use a recycled plastic sheet to contain the kit	Easy	High	A
4	Use a recycled plastic sheet to help condense dew/vapor and collect raindrops	Easy	High	A
5	Use gravitational force to allow water in	Easy	High	A
6	Use recycled newspaper sheets to transport moisture and control capillary	Easy	High	A
7	Use recycled materials	Easy	High	A
8	Reuse the tires for the next planting	Easy	High	A
9	Make a molded plastic cover to help collect moisture and rain	Hard	High	B
10	Make molded plastic plant cover to help protect against wind	Hard	High	B
11	Make deep holes to access deep land moisture	Hard	Low	D
12	Make large wind sheets to collect moisture from early down fog	Hard	Low	D
13	Add Nanoparticles to regulate irrigation	Hard	High	B
14	Use solar power to condense air moisture	Hard	High	B
15	Use cotton stuffing to collect moisture	Easy	Low	C
16	Use plastic valves to regulate water movement	Hard	High	B
17	Use water gel to retain moisture	Easy	Low	C
18	Use the metal body to store water	Hard	Low	D
19	Use shredded rubber tires to mix with soil to produce tiny water tanks	Easy	Low	C
20	Use type of plants that can quickly access the water table	Easy	High	A
21	Use drought-resistant plants	Easy	High	A
22	Use evergreen plants	Easy	High	A
23	Use plants that natural to desert habitat	Easy	High	A
24	Reduce the effect of wind through windscreens	Easy	High	A
25	Reduce sun heat through partial shading	Easy	High	A
26	Use recycled Waterboxx	Hard	High	B

Encouragement is given to the team in the VE method for using and targeting type A simple ideas. The unavailability of sufficient type A ideas brought attention to the class B ideas, and sufficient class A ideas were generated for our case.

### Alternative design

**Figure 8:**
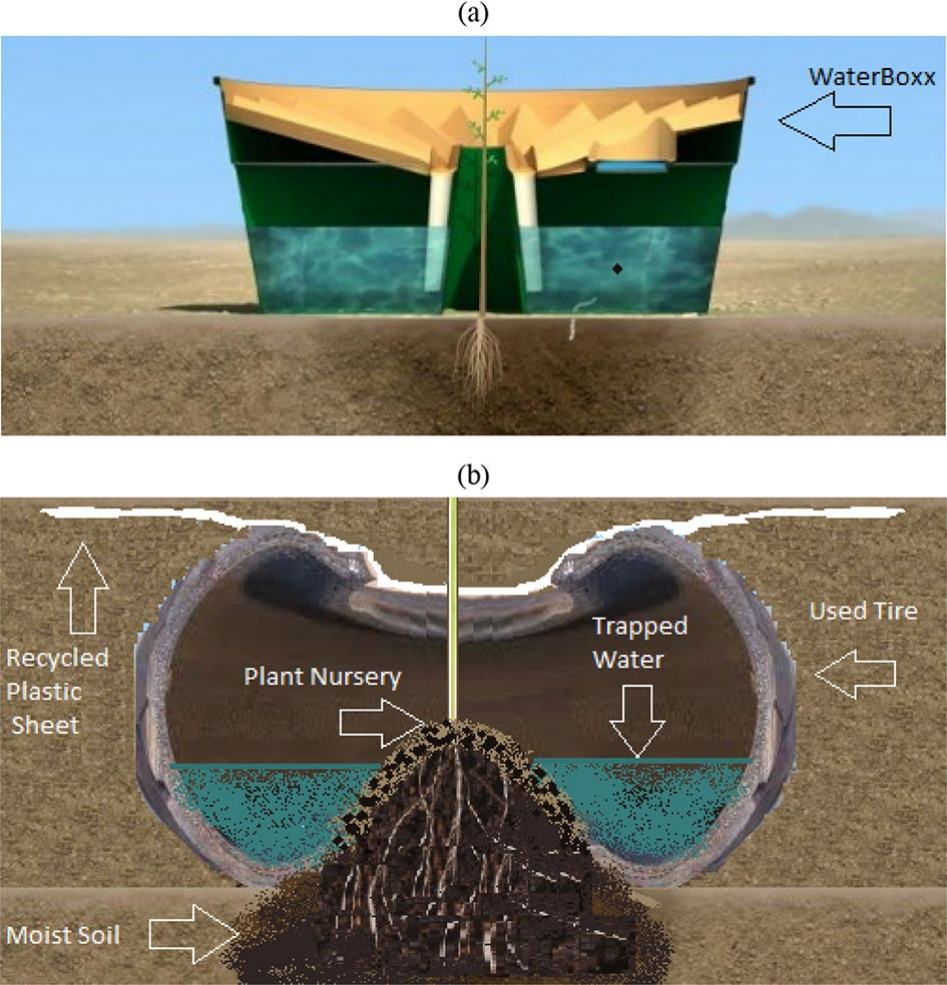
Waterboxx kit: (**a**) Original design, (**b**) Proposed alternative design.

The proposed alternative design procedures are recognized as the best known and effective model for our project. The rapid prototyping technique is considered a set of procedures employed to develop a scale prototype for the part of the original design or computer three-dimensional CAD design-based assembly^33,34^. Addictive layer manufacturing technique or three-dimensional printing is the mainly used simple manufacturing system for constructing a part or assembly^1,35^. Rapid prototyping was employed to generate prototype parts and replicas in early attempts in the late 1980s. Nowadays, it is employed in various applications. It is also implemented to manufacture high-quality parts without considering conventional overhead costs instead of small counts^36,37^. The recycled tires used in both the proposed new design and the Waterboxx design are demonstrated in Fig. 8. Due to the restrictions from the Patent Office, we couldn’t be able to disclose the detailed design specifications.

**Figure 9:**
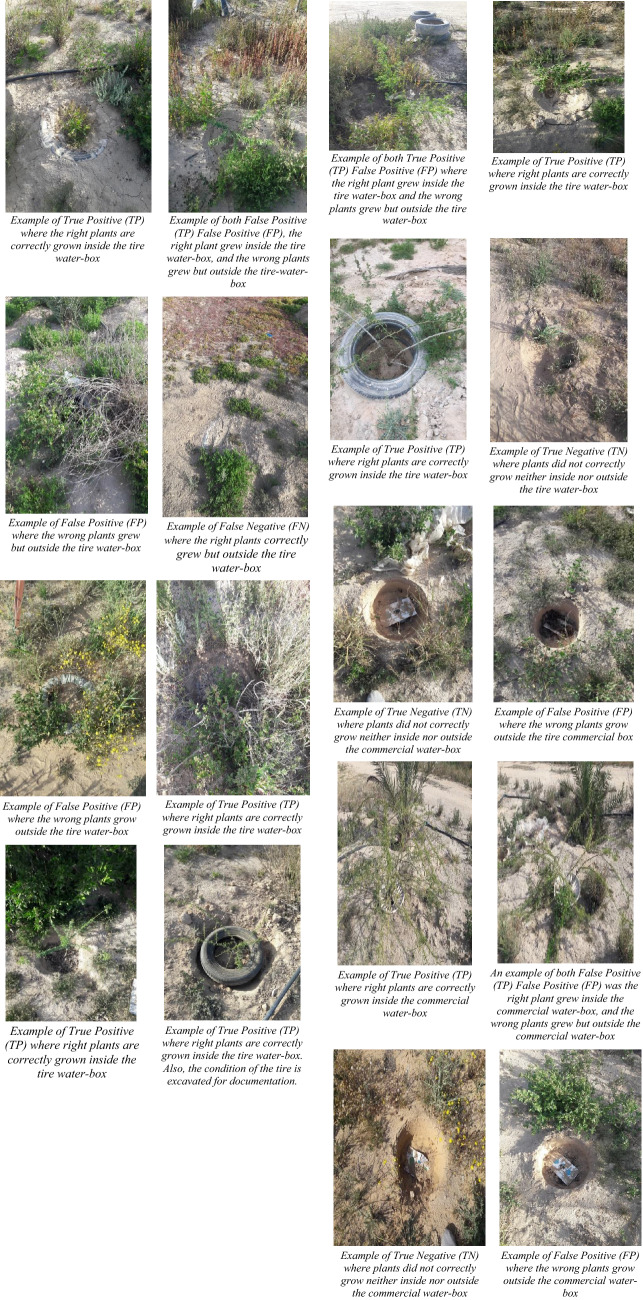
Examples of different scenarios of both designs.

The proposed models were implemented in potential laboratory conditions for testing and evaluating this alternative design before performing a full-fledge controlled environmental field test. The designs which have the most promising results are only tested and evaluated on the fields (Fig. 9).

### Results evaluation

Table 4 illustrates the comparison of the estimated cost for planting 1000 trees using both alternative design and normal Waterboxx for three years in Kuwaiti Dinars (1.0 US Dollar is equivalent to 0.303 Kuwaiti Dinars).

**Table 4:** Waterboxx & alternative design items’ cost for three-year life span.

Item	Waterboxx Cost (KD)	Alt. Design Cost (KD)
Trees	450	450
Fertilizer	900	900
Water	1095	1095
Labor	3240	3240
Kit	8000	2000
Total Cost	13,685	7685

The alternative design reduced costs and saved 43.84% of the total cost. These cost reduction does not pay attention to the intangible costs of plastics, paper, and recycled tires employed in the alternate model.

### Patent application

The proposed project’s design concept was put forward for patent registration in the USA under Patent file number: Docket No. 23588.79, “Utility Application for Planter System Using Waste Martials,” 2016. The patent registration was finalized and published on 18^th^ January 2018 with Publication No. US 2018/0014482 A1. The project is a self-irrigating planter system employing waste materials that promote plant growth in a protective environment.

### Abstract of the disclosure

This paper presented a self-irrigating planter system in a protective environment that was constructed using reused or repurposed Waste materials and promoted the growth of plants. A water reservoir buried in the soil included in the planter system was created using tire scrap with water storage by defining a hollow interior serving. For condensation, the water reservoir directly extends the sloped condensation skirt in the condensation funnel into the scrap tire’s center opening. The plant root system is covered by a root ball cover created using scrap newspaper. The soil of the covered root ball is protected by using a soil protection cover covered at the top of the root ball after burying the plant in the scarp tire’s center opening. The plant’s stem is protected from various environmental issues by extending a support tube. Water delivery to the reservoir is regulated by a valve. Scrap plastic is employed for creating most of these components.

## Site experiment perpetration

This venture employs a team with expertise in VM methodology for constructing a model for demonstrating an achievable substitute method to aid irrigation water rationing and fight desertification using recycled scrap tires that utilize domestic greeneries and plants for wind breaking the application. It is considered a revolutionary and cost-effective thought by employing recycled tires in the most popular Waterboxx kits. Waterboxx kits technology is considered a proven technology for reducing irrigation in planting bushes and scrubs in desert regions. The subsequent guidelines, focused in this research, are the following:

1. Environmental friendly and safe disposal of continually growing large dumpsters of the tire in the regions of Kuwait.
2. Preserving of priceless water assets and rainwater harvesting.
3. Employing futuristic methods to build the capacity of planting bushes and shrubs in Kuwaiti regions.

To evaluate the alternative design properly, the experiment is performed and evaluated on various test sites. To execute this, the team selected four different candidate sites of farms having an area of around 5000m^2^, as explained in Table 5.

**Table 5:** List of candidate test sites for proposed design.

Sites	# of Farms	Each Farm Area
Site # 1	4	5000 Square yard
Site # 2	4	5000 Square yard
Site # 3	4	5000 Square yard
Site # 4	4	5000 Square yard

A meeting is conducted with the research team and the representatives from the Kuwait Public Authority of Agriculture & Fishery (PAAF) to mention and discuss the various collaboration venues that are feasible for conducting this experiment. PAAF very much supported this venture, and they assigned a few persons to identify some sites to take the region for experimentation. The research team checked various websites and collected water and soil from the proposed sites to study and determine those places.

**Figure 10:**
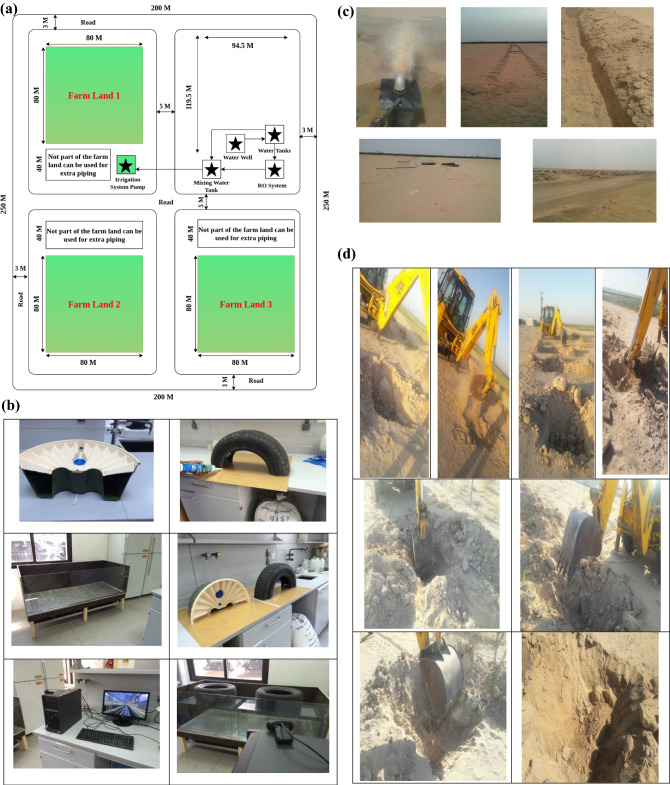
(**a**) A proposed structure of a test site. (**b**) Various pictures of the box are manufactured and designed for a controlled test environment. (**c**) Various pictures of the land being prepared and ready for use as a field test environment. (**d**) Various pictures of the test site preparation land used as a field test environment.

The test is conducted in mainly two ways: in an environmentally controlled laboratory and the field. The test in the lab is conducted through accurate observation. Next, the test is performed in a real-world environmental condition, demonstrated in Fig. 10a. These two test scenarios are parallelly carried out. Figure 10b illustrates the container box designed for testing in one of the laboratories having a controlled test environment inside Kuwait University. The test box contains actual soil collected from the test location is an actual demonstration of the test site. Figure 10c and d depict the real-world test locations. The irrigation water used in the test is collected from the wells in the test locations.

Various test sites are considered to evaluate the quality of the newly presented alternative model with scrap tires (Tire-Waterboxx) for the Groasis Waterboxx approach. The number Tire-Waterboxxes would be examined and converted to a from each test site 2 × 2 contingency matrix, as shown in Table 6 for each test site. The contingency matrix consists of four parameters, namely TP (True Positive), TN (True Negative), FP (False Positive), and FN (False Negative). The explanations for these parameters are the following:

- TN: The number of nurseries correctly grown to not trees
- FP: The number of nurseries incorrectly grown to trees
- FN: The number of nurseries incorrectly grown to not trees
- TP: The number of nurseries with perfectly grown trees

**Table 6:** Contingency table.

		Actual Plants planted	
		0	1
Plants grown to Trees	0	TN	FN
	1	FP	TP

To evaluate the effectiveness of the new model of the tire-Waterboxx method, conventional *recall* and *precision* metrics were proposed by Olson and Delen^36^. Equation 1 explains both *precision* and *recall* evaluation metrics.

1 $$precision = \frac{TP}{{TP + FP}}{\text{ and }}recall = \frac{TP}{{TP + FN}} \, {.}$$

The alternative design reduced costs and saved 43.84% of the total cost. These cost reduction does not pay attention to the intangible costs of plastics, paper, and recycled tires employed in the alternate model (Table 4).

### Calculation of the function measure

The quality of the newly presented Waterboxx model utilizing tires is to be measured. Diversified farms at various locations are considered for assessing the model quality, and Tire-Waterboxes would be experimented with for planting trees on each farm. Table 7 depicts the generated contingency table having the order 2 × 2 based on this setting, and it consists of four parameters, namely TP (True Positive), TN (True Negative), FP (False Positive), and FN (False Negative). The interpretations of these parameters in this context are explained as follows.

- TN: The count of plants that did not flourish to trees (neither inside nor outside the water-box of any type).
- FP: The count of plants that either grew to trees inside the water-box of the wrong type (FP1) or grown outside the water-box of the wrong type (FP2)
- FN: The count of plants that flourished to trees of the right type but outside the water-box,
- TP: The count of plants successfully grown to trees or the right type inside the water-box.

**Table 7:** Contingency table for tire-water-box.

		Actual Plants planted	
		0	1
Plants grown to Trees	0	11.70%	6.30%
	1	16.00%	58.70%

The evaluation metrics used for estimating the effectiveness of tire-water-box plantation are precision and recall, as explained in Eq. 1.

F-Measure^38^ is also an evaluation metric estimated based on recall and precision and is considered a single measure for expressing classification quality. It is estimated as the harmonic mean of recall and precision, as explained in Eq. 2.

2 $$Fmeasure = \frac{2 \times precision \times recall}{{precision + recall}}.$$

The detailed evaluation results using various metrics precision, recall, and F-Measure are listed in Table 8.

**Table 8:** Results of proposed tire-water-box in Lots.

Lot	TN (%)	FP			FN (%)	TP (%)	Precision (%)	Recall (%)	Fmeasure (%)
		FP1 (%)	FP2 (%)	∑ FP1,2 (%)					
Lot1	10	9	5	14	5	55	79.70	91.70	85.30
Lot2	13	8	6	14	8	59	80.80	88.10	83.60
Lot3	12	12	8	20	6	62	75.60	91.30	82.70

Average F-measure of proposed tire-water-box in Lots = 83.9%.

### Contingency table

In a two-way classification on a specific data set, the classifier Classifies the data into these two classes, either positive or negative. We get two counts: The count of positively classified data and the count of negatively classified data, the sum of these counts gets the count of the entire data set. For evaluating the performance of this classifier, we need a reference classification that should be ideally classified, but in practice, employed the results of a gold standard set. A 2 × 2 Contingency table is formed with these two classifiers’ outputs for cross tabulating the data. Four parameters can summarize the contingency table. Usually, these parameters are scale-invariant Since there is no variation in the output by scaling each parameter by the same factor. For making these statistics independent of the size of the population, ratios of homogeneous functions such as linear or quadratic can be used.

Summing up these parameters generates marginal and grand totals. The sum of these four parameters, true positive, true negative, false positive, and false negative, produces the entire set. It is possible to add up the tables horizontally and vertically to produce different statistics. The horizontal addition of the first row yields the number of test positives, and the second row produces the counts of test negatives. Similarly, the vertical addition of the first column gives the counts of true positives and the second column shows the true negatives. The fraction of the four values in the given table with the row or column marginal total should obtain the statistics of basic marginal ratio: generates two supplementary tables of order 2 × 2 for eight ratios in total.

It should be noted that these ratios are viewed as complementary pairs, and the sum of each of these pairs is 1. So, we can summarize these derived tabled into a couple of two numbers along with their complements. Further statistics can be obtained by ratios of these ratios or more complicated functions.

The proposed methodology was tested on three farm lots to perform its evaluation. It is predicted, after extensive experimentations, the proposed technique produces the following quality results.

### Alternatives function evaluation

In the following Tables 9 and 10, we present the result of our findings.

**Table 9:** Results of commercial Waterboxx in Lots.

Lot	TN (%)	FP			FN (%)	TP (%)	Precision (%)	Recall (%)	F-measure (%)
		FP1 (%)	FP2 (%)	∑ FP1,2 (%)					
Lot1	17	8	1	9	0	74	89.20	100	94.30
Lot2	19	7	2	9	0	72	88.90	100	94.10
Lot3	12	10	1	11	1	76	87.40	98.70	92.70

Average F-measure of commercial Waterboxx in Lots = 93.7%.

**Table 10:** Contingency table for commercial Waterboxx.

		Actual Plants planted	
		0	1
Plants grown to Trees	0	16.0%	0.3%
	1	9.7%	74.0%

Note that the difference of commercial Waterboxx over tire-water-box in F-measure is about 10.5%. However, the proposed tire-water-box has a higher impact in terms of creating microenvironments for other plants to grow and prosper (higher FP).

Figure (Fig. 9) demonstrates examples of the different scenarios of both designs.

### Design value index

Value index calculations in Value Engineering Methodology is calculated according to the following formula:

$$Value = (Function*Quality)/Cost,V = (F*Q)/C$$

**F** is computed according to the F-measure, **Q** is the quantification of the quality, **C** is the total life cycle cost.

To compute the value index, it is customary to do a weighted value evaluation exercise. Function cost worth analysis is termed as value index, which measures the cost vs. worth of the proposed model^39^. Many of the previous researches employed and evaluated the value indexes and their relationships with the model^21,39,40,42–47^. Table 11 computes the value of both the original commercial Waterboxx and the new proposed tire-water-box.

**Table 11:**
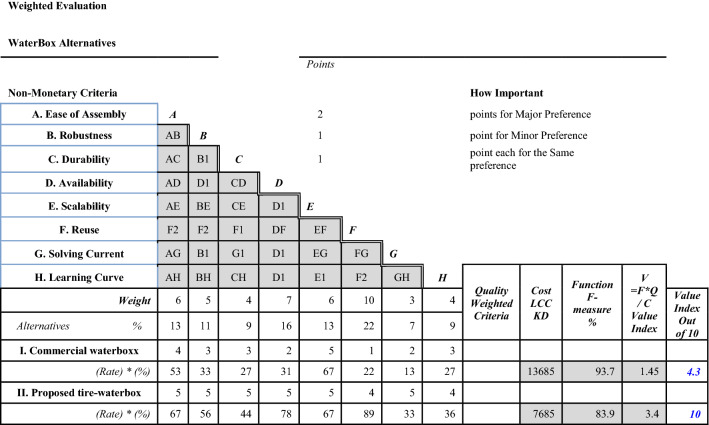
Value index calculation of original and new designs.

It is clear that the value index of the proposed tire-water-box is much higher (double) than the value of commercial Waterboxx.

### Environmental impact

The original design was deployed in 250 kits in the selected area of the experiment. A total of 3*250 tires were deployed in the three plots of the selected site for experimentation of the final proposed design. The environmental impact on the soil; and water was analyzed by conduction pre-field test and post field test soil and water analysis. The results showed that both the commercial Waterboxx and the proposed tire-water-box did not significantly impact the soil and water quality. Residues for both kits on soil and water were within acceptable ranges after almost three years of usage.

#### Soil test results

Table 12 summarizes the soil test of original and new designs after the field testing.

**Table 12:** Summary of soil test results of original and new designs.

Soil Tests	2015 results			2018 results						Difference %		
	Original (1)			Commercial WaterBoxx (2)			Proposed Tire-waterbox (3)			∆_1,2_	∆_1,3_	∆_2,3_
	Min	max	Average	min	max	Average	min	max	average			
Ammonia (mg/L)	0.01	10.48	2.52	3.45	3.45	3.45	1.27	11.03	6.33	36.9	151.2	114.3
Alkalinity (total)	95.78	484.32	258.47	1408.45	1408.45	1408.45	2418.6	3717.24	3184.72	444.9	1132.1	687.2
Nitrite/Nitrate (NO2/NO3 mg/kg)	2.16	9.84	4.74	12.53	12.53	12.53	13.32	24.56	18.12	164.3	282.3	117.9
Silicate (Si mg/kg)	3.17	12.49	8.31	136.48	136.48	136.48	106.47	123.09	114.65	1542.4	1279.7	−262.7
TPH in Soil (ug/L)	< 0.10	< 0.10	< 0.10	< 0.10	< 0.10	< 0.10	< 0.10	< 0.10	< 0.10	0.0	0.0	0.0
Extractable Organic Matter (EOM mg/kg dry weight)	< 400	1,533	416.5	< 400	< 400	< 400	< 400	< 400	< 400	−4.0	−4.0	0.0
pH	8.01	8.07	8.04	8.03	8.03	8.03	7.72	7.96	7.84	−0.1	−2.5	−2.4
Moisture (moist %)	1.24	5.21	3.43	1.88	1.88	1.88	4.71	5.76	5.36	−45.2	56.3	101.5

The tire-water-box has achieved a much better average soil moisture than the commercial Waterboxx, and it also has higher total Alkalinity and almost the exact value of pH. The soil Nitrite/Nitrate and Ammonia contents also were elevated in the new design. On the other hand, the newly designed tire water-box had lowered silicate contents in the soil as while the organic matters and TPH remain the same for both solutions where they do not seem to affect this in the surrounding soil.

#### Water test results

Table 13 summarizes the water test of original and new designs after the field testing.

**Table 13:** Summary of water test results of original and new designs.

Water tests	2015 results			2018 results						Difference %		
	Original (1)			Commercial WaterBoxx (2)			Proposed Tire-waterbox (3)			∆_1,2_	∆_1,3_	∆_2,3_
	min	min	max	average	max	average	min	max	average			
Ammonia (mg/L)	0.01	0.01	0.01	0.01	0.01	0.01	0.01	0.01	0.01	0.0	0.0	0.0
Alkalinity (total)	139.04	139.04	139.04	106.58	106.58	106.58	130.5	163.13	147.90	−23.3	6.4	29.7
Nitrite/nitrate (NO2/NO3 mg/kg)	47.30	47.30	47.30	0.45	0.45	0.45	4.52	17.90	9.33	−99.0	−80.3	18.8
Silicate (Si mg/kg)	11.90	11.90	11.90	16.50	16.50	16.50	27.50	34.10	30.60	38.7	157.1	118.5
TPH in water (ug/L)	< 0.40	< 0.40	< 0.40	1.52	1.52	1.52	0.60	1.27	0.88	280.0	120.0	−160.0
pH	8.05	8.17	8.11	7.49	7.49	7.49	7.96	8.02	8.00	−7.6	−1.4	6.3

The tire-box has achieved higher water average Alkalinity than the commercial waterboxx. The water Nitrite/Nitrate and Silicate contents also were elevated in the new design, whereas the TPH contents in the water were lowered. The pH value of water in both solutions remains almost the same.

Simulation of all test sites’ conditions of Kuwait is well performed in the laboratory, and the changes that appeared under the soil are analyzed and documented. This will be performed by video recording the plant life cycle with a computer workstation and a side view using both the testing methodology next to the Waterboxx equipment and alternate models. It also considered the available software packages for simulating water or soil behavior.

## Concluding remarks

The results accomplished using this proposed model macadamized a baseline for developing a comprehensive and more aggressive national scheme for water resource optimization and fighting against desertification. The proposed alternative design is a cost-effective and environmentally friendly model based on VE methodology by employing recycled tires and supporting the environment. This design develops a self-irrigating planter system using recycled waste materials and supports the plant’s growth in a protective environment. A water reservoir is also included in this planter system buried in the soil and made from tire scrap; a hollow interior serving defined in it is used as water storage. Tire scrap, plastic, and newspapers are employed for making most of these components^41^.

The proposed alternative design has been proved through the experiments performed that this model has the effectiveness of similar or superior with much lesser expenditure as a commercial model. The proposed model reduced the Cost with VE redesign and saved 43.84% of the overall cost. The overall cost reduction of the alternate design may be intensified by analyzing and calculating intangible costs such as waste recycling. The significance of Value Engineering technology in developing innovative ideas for cost reduction is also proved in this study.
